# Fractional Euler numbers and generalized proportional fractional logistic differential equation

**DOI:** 10.1007/s13540-022-00044-0

**Published:** 2022-05-27

**Authors:** Juan J. Nieto

**Affiliations:** CITMAga, Departamento de Estatística, Análise Matemática e Optimización, Universidade de Santiago de Compostela, 15782 Santiago de Compostela, Spain

**Keywords:** Logistic differential equation, Fractional calculus, Generalized proportional fractional integral, Euler numbers, Euler fractional numbers, 34A08 primary, 11B68

## Abstract

We solve a logistic differential equation for generalized proportional Caputo fractional derivative. The solution is found as a fractional power series. The coefficients of that power series are related to the Euler polynomials and Euler numbers as well as to the sequence of Euler’s fractional numbers recently introduced. Some numerical approximations are presented to show the good approximations obtained by truncating the fractional power series. This generalizes previous cases including the Caputo fractional logistic differential equation and Euler’s numbers.

## Introduction

The logistic ordinary differential equation $u'(t)=u(t)\cdot [1-u(t)]$ appears in many contexts and have different applications in physics [3, 8, 9, 11], medicine [17], economy [16], and even to study the evolution of the COVID-19 epidemic [13, 14].

The solution, for a given initial condition $u(0)=u_0,$ is

$$\begin{aligned} u(t)=\frac{u_0}{u_0+(1-u_0)e^{-t}}. \end{aligned}$$

For $$u_0=1/2 , $$ we have the classical logistic function

$$\begin{aligned} l(t)=\frac{1}{1+e^{-t}}. \end{aligned}$$

Different versions and generalizations of the logistic equation have been considered and, in particular, the fractional versions of the logistic differential equation [2–5, 12, 15].

For example, a fractional version has been studied:

1.1 $$\begin{aligned} D^{\alpha }x(t)=x(t)\cdot [1-x(t)] \end{aligned}$$

with $\alpha \in (0,1)$ and $D^{\alpha }$ the Caputo fractional derivative [7]. Although an analytical expression for the solutions is not known, it has been solved using different techniques such Euler’s numbers [4, 6], implicit solutions [10] or fractional power series [3].

We study a new generalization of the fractional differential equation (1.1) that includes as a particular case the Caputo fractional logistic differential equation.

Moreover, we use fractional generalized proportional derivative having a singular kernel.

We introduce a novel class of Euler’s numbers, the generalized proportional fractional Euler numbers. We recall that Euler’s polynomials and Euler’s numbers are related to the Riemann’s zeta function and to the logistic function [3]. The relevance is apparent due to the importance of solving the famous Riemann Hypotheses.

This paper is organized as follows. In the next section we introduce the generalized proportional fractional calculus with its basic concepts and properties. Then, it is solved a simple linear fractional differential equations to motivate our technique in order to solve a generalized fractional logistic differential equation. Finally in the last section, we present the generalized proportional fractional Euler’s numbers. Euler’s numbers appear in connection to the most important function in mathematics: the zeta function.

## Generalized proportional calculus

Let $T >0,$ $\alpha >0$ be the order of the fractional integral and $\rho \in (0,1]$ be the proportion. For a function $u \in L^1(0,T)$ we define the generalized proportional integral of the function *u* as

$$\begin{aligned} I^{\alpha ,\, \rho } u(t) = \frac{1}{\rho ^\alpha \cdot \varGamma (\alpha )} \int _{0}^{t}{e^{\frac{\rho -1}{\rho }(t-s)}(t-s)^{\alpha -1}u(s)ds}, \, t \in [0,T]. \end{aligned}$$

The corresponding Caputo generalized proportional fractional derivative for a function $u \in L^1(0,T)$ such that $u \in AC[0,T]$ is defined as [1]

$$\begin{aligned} ^C\mathcal{{D}}^{\alpha , \rho } u(t)= & {} [ (I^{1-\alpha , \rho } \circ \mathcal{{D}}^{1, \rho }) u ](t) \\= & {} \frac{1}{\rho ^{1-\alpha } \cdot \varGamma (1-\alpha )} \int _{0}^{t}{e^{\frac{\rho -1}{\rho }(t-s)}(t-s)^{-\alpha } \, \mathcal{{D}}^{1, \rho } u(s)ds} \end{aligned}$$

where

$$\begin{aligned} \mathcal{{D}}^{1, \rho } u(t) = (1-\rho ) u(t) + \rho u'(t). \end{aligned}$$

We note that for $\rho =1$ we obtain the classical Caputo fractional derivative [7]:

$$\begin{aligned} ^C\mathcal{{D}}^{\alpha , 1} u(s) = \frac{1}{ \varGamma (1-\alpha )} \int _{0}^{t}{(t-s)^{-\alpha } \, u'(s) \, ds} := \, ^CD^{\alpha }u(t). \end{aligned}$$

We recall that [1]

$$\begin{aligned} (I^{\alpha , \rho } (^C\mathcal{{D}}^{\alpha , \rho }u))(t) = u(t) - u(0) \cdot e^{\frac{\rho -1}{\rho }t}. \end{aligned}$$

Also, for $\beta >0$, consider the function

$$\begin{aligned} \xi _{\rho ,\beta } (t) = e^{\frac{\rho -1}{\rho }t}\cdot t^{\beta -1} , \end{aligned}$$

then

$$\begin{aligned} (I^{\alpha , \rho } \xi _{\rho ,\beta }) (t) = \frac{\varGamma (\beta )}{\rho ^\alpha \cdot \varGamma (\beta +\alpha )} \cdot e^{\frac{\rho -1}{\rho }t}\cdot t^{\beta -1+\alpha } = \xi _{\rho ,\beta +\alpha }(t) . \end{aligned}$$

We now study some differential equations under this generalized fractional calculus. Indeed, consider the nonlinear differential equation of the type

2.1 $$\begin{aligned} ^C\mathcal{{D}}^{\alpha , \rho } u(t) = f(t,u(t)) \end{aligned}$$

with the initial condition

$$\begin{aligned} u(0)=u_0. \end{aligned}$$

Here $f:[0,T]\times \mathbf{{ R}} \rightarrow \mathbf{{R}}$ is a nonlinear function satisfying appropriate conditions.

## Linear generalized proportional differential equations

Let $\sigma \in L^1(0,T)$ so that the corresponding generalized proportional integral of $\sigma$ exists.

We begin with the simple case

$$\begin{aligned} ^C\mathcal{{D}}^{\alpha , \rho } u(t) =\sigma (t) , \, \, \, u(0)=u_0. \end{aligned}$$

By applying the generalized proportional fractional integral, we have

$$\begin{aligned} u(t) - u_0 \cdot e^{\frac{\rho -1}{\rho }t} + \frac{1}{\rho ^\alpha \varGamma (\alpha )} = \int _{0}^{t}{e^{\frac{\rho -1}{\rho }(t-s)}(t-s)^{\alpha -1} \sigma (s)ds}, \, t \in [0,T]. \end{aligned}$$

Therefore,

$$\begin{aligned} u(t) = u_0 \cdot e^{\frac{\rho -1}{\rho }t} + \frac{1}{\rho ^\alpha \varGamma (\alpha )} \int _{0}^{t}{e^{\frac{\rho -1}{\rho }(t-s)}(t-s)^{\alpha -1} \sigma (s)ds}, \, t \in [0,T]. \end{aligned}$$

Now, for $\lambda \in \mathbf{R}$, let us study the following linear differential equation

3.1 $$\begin{aligned} ^C\mathcal{{D}}^{\alpha , \rho } u(t) =\lambda u (t) , \, \, \, u(0)=u_0. \end{aligned}$$

The solution is known [1]

$$\begin{aligned} u(t)= u_0 e^{\frac{\rho -1}{\rho }t} \mathcal{{E}}_\alpha \left( \lambda \left( \frac{t}{\rho }\right) ^\alpha \right) , \end{aligned}$$

where $\mathcal{{E}}_{\alpha }$ is the classical Mittag-Leffler function defined for any $z \in \mathbf{{C}}$ as

$$\begin{aligned} \mathcal{{E}}_\alpha (z) = \sum _{n=0}^{\infty }{\frac{z^n}{\varGamma (\alpha n +1)}} . \end{aligned}$$

We now re-obtain this solution as a fractional power series. Moreover, this will serve as a clear introduction to our methodology. Indeed, take $r=\frac{\rho -1}{\rho }$ and let us assume that the solution is given formally as the following fractional power series

$$\begin{aligned} u(t) = e^{rt} \sum _{n=0}^{\infty }{a_n (t^\alpha )^n.} \end{aligned}$$

Thus, formally,

$$\begin{aligned} I^{\alpha ,\rho }u(t) = \sum _{n=0}^{\infty }{a_n I^{\alpha ,\rho }(e^{rt}t^{\alpha n}}) = \sum _{n=0}^{\infty }{a_n \frac{\varGamma (\alpha n + 1)}{\rho ^\alpha \varGamma (\alpha (n+1)+1)}e^{rt}t^{\alpha (n+1)}} \end{aligned}$$

and

$$\begin{aligned} e^{rt} \sum _{n=0}^{\infty }{a_n (t^\alpha )^n} = u_0 e^{rt} + \lambda \sum _{n=0}^{\infty }{a_n \frac{\varGamma (\alpha n + 1)}{\rho ^\alpha \varGamma (\alpha (n+1)+1)}e^{rt}t^{\alpha (n+1)}}. \end{aligned}$$

Equivalently,

$$\begin{aligned} e^{rt} \sum _{n=0}^{\infty }{a_n (t^\alpha )^n} = u_0 e^{rt} +\lambda \sum _{n=1}^{\infty }{a_{n-1} \frac{\varGamma (\alpha ( n-1) + 1)}{\rho ^\alpha \varGamma (\alpha n+1)}e^{rt}t^{\alpha n }}. \end{aligned}$$

Identifying the coefficients, we get $a_0 = u_0$ and the recurrence formula

$$\begin{aligned} a_n = \lambda a_{n-1} \frac{\varGamma (\alpha ( n-1) + 1)}{\rho ^\alpha \varGamma (\alpha n+1)} \, ,\, \, n \ge 1. \end{aligned}$$

This implies that

$$\begin{aligned} a_n = a_0 \left( \frac{\lambda }{\rho ^\alpha } \right) ^n \frac{1}{\varGamma (\alpha n +1)} \end{aligned}$$

so that

$$\begin{aligned} u(t)= u_0 e^{rt} \sum _{n=0}^{\infty }{\left( \frac{\lambda }{\rho ^\alpha } \right) ^n} \frac{t^{\alpha n}}{\varGamma (\alpha n+1)} = u_0 e^{\frac{\rho -1}{\rho }t} \mathcal{{E}}_\alpha (\lambda (\frac{t}{\rho })^\alpha ) . \end{aligned}$$

**Fig. 1:**
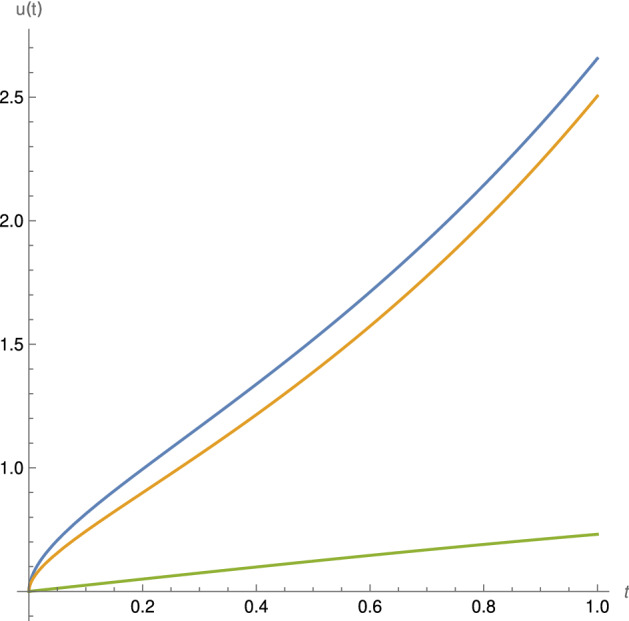
Solutions of the linear generalized proportional fractional differential equations (3.1) with $\lambda =1$ for the initial condition $x_0= 1/2$ and $\alpha =1/2$. For $\rho =1/2$ in blue. For $\rho =1$ the Caputo fractional differential equation (3.2) in the middle (orange) and the classical logistic function below (green)

See Fig. 1. For $\rho =1$, that is $r=0$ we have the classical fractional Caputo equation

3.2 $$\begin{aligned} ^C D^\alpha u = \lambda u \, , \, \, u(0)= u_0 \end{aligned}$$

whose solution is indeed given by

$$\begin{aligned} u(t) = u_0 \, \mathcal{{E}}_\alpha (\lambda t^\alpha ). \end{aligned}$$

## Logistic generalized proportional differential equations

We now consider a logistic-type equation corresponding to the nonlinear equation (2.1) with

$$\begin{aligned} f(t,u(t))= \lambda u(t) - \mu e^{-rt}u^2(t) \, , \end{aligned}$$

where $\lambda , \, \mu \in R$ and $r= \frac{\rho -1}{\rho } \, ,$ that is, the logistic fractional generalized proportional differential equation

4.1 $$\begin{aligned} ^C\mathcal{{D}}^{\alpha , \rho } u(t) = \lambda u(t) - \mu e^{-rt}u^2(t) \, , \, \, u(0)= u_0. \end{aligned}$$

For $\mu =0$ we have the previous linear equation (3.1).

In the case $\rho =1$, that is, $r=0$, we obtain the following Caputo fractional logistic differential equation

$$\begin{aligned} ^C D^\alpha u(t) = \lambda u(t) - \mu u^2(t) \end{aligned}$$

that has been solved recently [3].

As for the linear situation, we assume that

4.2 $$\begin{aligned} u(t) = e^{rt} \sum _{n=0}^{\infty }{a_n (t^\alpha )^n.} \end{aligned}$$

Then, using the Cauchy product we get

$$\begin{aligned} e^{-rt}u^2(t) = e^{rt} \sum _{n=0}^{\infty }{b_n (t^\alpha )^n} \, , \, \, b_n = \sum _{k=0}^{n}{a_{n-k} \cdot a_k}. \end{aligned}$$

Therefore,

$$\begin{aligned} e^{rt} \sum _{n=0}^{\infty }{a_n (t^\alpha )^n}= & {} u_0 e^{rt} +\lambda \sum _{n=1}^{\infty }{a_{n-1} \frac{\varGamma (\alpha ( n-1) + 1)}{\rho ^\alpha \varGamma (\alpha n+1)}e^{rt}t^{\alpha n }}\\&-\mu \sum _{n=1}^{\infty }{b_{n-1} \frac{\varGamma (\alpha ( n-1) + 1)}{\rho ^\alpha \varGamma (\alpha n+1)}e^{rt}t^{\alpha n }}. \end{aligned}$$

Identifying the coefficients corresponding to the powers of $t^\alpha$ , we get

$$\begin{aligned} a_0 = u_0 \end{aligned}$$

and for $n \ge 1$, the recurrence relation

$$\begin{aligned} a_n = \lambda a_{n-1} \frac{\varGamma (\alpha ( n-1) + 1)}{\rho ^\alpha \varGamma (\alpha n+1)} - \mu b_{n-1} \frac{\varGamma (\alpha ( n-1) + 1)}{\rho ^\alpha \varGamma (\alpha n+1)} \end{aligned}$$

or

4.3 $$\begin{aligned} a_n(\alpha ,\rho ,\lambda ,\mu ) = \frac{\varGamma (\alpha ( n-1) + 1)}{\rho ^\alpha \varGamma (\alpha n+1)} \left( \lambda a_{n-1}- \mu b_{n-1} \right) . \end{aligned}$$

Taking the initial condition $u(0)=1/2$ so that $a_0 = 1/2.$ Thus, for example,

$$\begin{aligned} a_1= & {} \frac{\rho ^{-\alpha } \left( \frac{\lambda }{2}-\frac{\mu }{4}\right) }{\varGamma (\alpha +1)},\\ a_2= & {} \frac{\rho ^{-\alpha } \varGamma (\alpha +1) \left( \frac{\lambda \rho ^{-\alpha } \left( \frac{\lambda }{2}-\frac{\mu }{4}\right) }{\varGamma (\alpha +1)}-\frac{\mu \rho ^{-\alpha } \left( \frac{\lambda }{2}-\frac{\mu }{4}\right) }{\varGamma (\alpha +1)}\right) }{\varGamma (2 \alpha +1)} \end{aligned}$$

and

$$\begin{aligned} \frac{\varGamma (3 \alpha +1)}{\rho ^\alpha \varGamma (2 \alpha +1)} \cdot a_3= & {} \frac{\lambda \rho ^{-\alpha } \varGamma (\alpha +1) \left( \frac{\lambda \rho ^{-\alpha } \left( \frac{\lambda }{2}-\frac{\mu }{4}\right) }{\varGamma (\alpha +1)}-\frac{\mu \rho ^{-\alpha } \left( \frac{\lambda }{2}-\frac{\mu }{4}\right) }{\varGamma (\alpha +1)}\right) }{\varGamma (2 \alpha +1)}\\&-\mu \left( \frac{\rho ^{-2 \alpha } \left( \frac{\lambda }{2}-\frac{\mu }{4}\right) ^2}{\varGamma (\alpha +1)^2}+\frac{\rho ^{-\alpha } \varGamma (\alpha +1) \left( \frac{\lambda \rho ^{-\alpha } \left( \frac{\lambda }{2}-\frac{\mu }{4}\right) }{\varGamma (\alpha +1)}-\frac{\mu \rho ^{-\alpha } \left( \frac{\lambda }{2}-\frac{\mu }{4}\right) }{\varGamma (\alpha +1)}\right) }{\varGamma (2 \alpha +1)}\right) . \end{aligned}$$

**Fig. 2:**
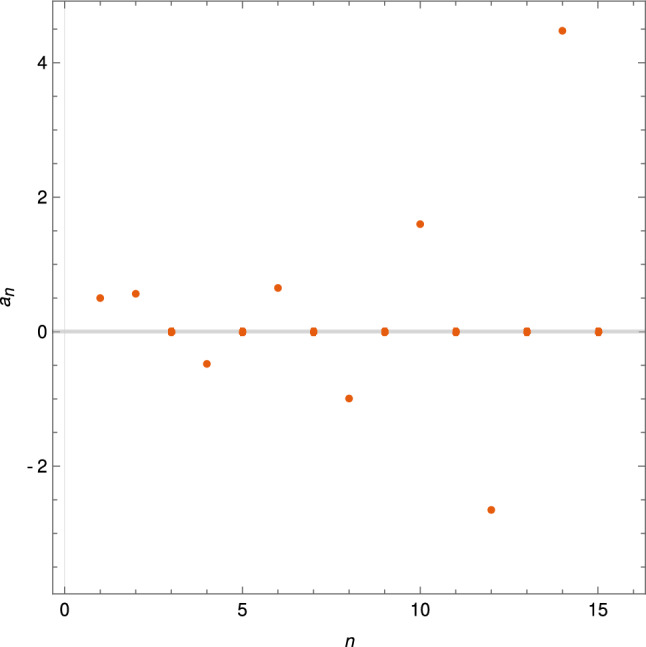
Coefficients for the generalized proportional fractional logistic equation for $\alpha =1/2$ and $\rho =1/4.$

For example, for $\alpha =1/2$, $\rho =1/4$ and $\lambda =\mu =1$ we have the following values of $a_n(1/2,1/4)$ for $n=0,1,\dots ,15$ (See Fig. 2)

$$\begin{aligned}&\frac{1}{2},\frac{1}{\sqrt{\pi }},0,-\frac{8}{3 \pi ^{3/2}},0,\frac{512}{45 \pi ^{5/2}},0,-\frac{4096}{75 \pi ^{7/2}},0,\frac{8388608}{30375 \pi ^{9/2}},0,-\frac{2751463424}{1913625 \pi ^{11/2}},\\&0,\frac{43825846288384}{5746615875 \pi ^{13/2}},0,-\frac{168366428854943744}{4108830350625 \pi ^{15/2}} \dots \end{aligned}$$

The solution of the classical ordinary differential equation

$$\begin{aligned} u'=u(1-u) \end{aligned}$$

with the initial condition

$$\begin{aligned} u(0)=1/2 \end{aligned}$$

is the logistic function

$$\begin{aligned} u(t)=\frac{1}{1+e^{-t}}= \sum _{n=0}^{\infty }{a_nt^n}. \end{aligned}$$

In this case, $\rho =1 \, , \, \, \alpha =1 \, , \, \, \lambda = \mu =1$ and the recurrence relation is

$$\begin{aligned} a_n = \frac{1}{n} \left[ a_{n-1}- \sum _{k=0}^{n-1}{a_{n-1-k}a_k }\right] . \end{aligned}$$

**Fig. 3:**
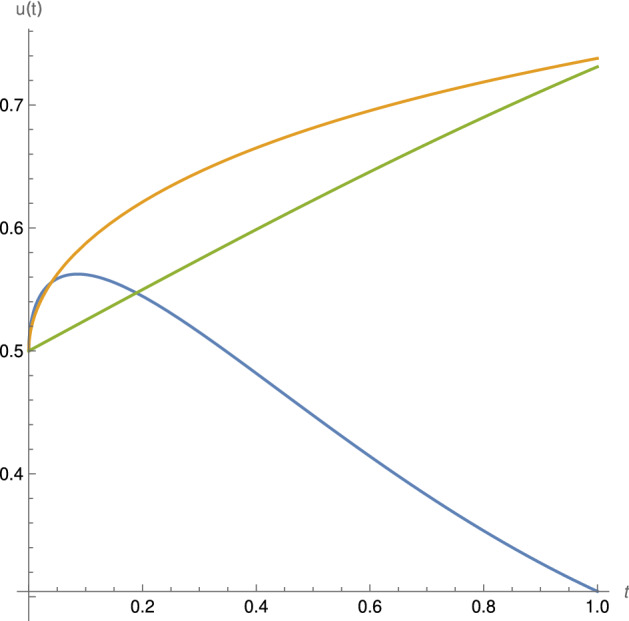
Approximate solution $p_m(t) \, , \, \, m=10 ,$ of the logistic generalized proportional fractional differential equations (4.1) for the initial condition $x_0= 1/2$ and $\alpha =1/2$. For $\rho =1/2$ in blue. For $\rho =1$ the corresponding approximate solution $p_{10}(t)$ of the Caputo fractional logistic differential equation (3.2) (orange) and the classical logistic function below (green)

The solution of new the logistic equation (4.1) is given by (4.2) and can be approximated by (See Fig. 3)

$$\begin{aligned} p_m(t) = e^{rt} \sum _{n=0}^{m}{a_n (t^\alpha )^n} \, , \, \, m \ge 1. \end{aligned}$$

## Generalized proportional Euler numbers

We recall the Euler polynomials defined as

$$\begin{aligned} \frac{2 e^{xt}}{e^t + 1} = \sum _{n=0}^{\infty }{E_n(x)\frac{t^n}{n!}} \, , \, \, |t|<\pi . \end{aligned}$$

Taking $x=1$ we derive

$$\begin{aligned} \frac{1}{1+e^{-t}}=\sum _{n=0}^{\infty }{\frac{1}{2}E_n(1)\frac{t^n}{n!}} = \frac{1}{2}+\frac{1}{4}t-\frac{1}{48}t^3+\frac{1}{480}t^5+ \dots = \sum _{n=0}^{\infty }{a_n t^n} , \end{aligned}$$

where

$$\begin{aligned} a_n = \frac{1}{n} [ a_{n-1}- \sum _{k=0}^{n-1}{a_{n-1-k}a_k }] \, , \, \, n\ge 1. \end{aligned}$$

This logistic function is the solution of the logistic problem

$$\begin{aligned} u'=u(1-u) \, , \, \, u(0)=\frac{1}{2}. \end{aligned}$$

It is well-known that the coefficients $a_n$ are related to the Euler numbers $E_n$ by

$$\begin{aligned} a_n=\frac{E_n}{2\cdot n!}. \end{aligned}$$

For a given $a_0 \in R$ and in view of (4.3), we define the generalized proportional fractional Euler numbers by the recurrence relation

5.1 $$\begin{aligned} a_n(\alpha ,\rho ,\lambda ,\mu ) = \frac{\varGamma (\alpha ( n-1) + 1)}{\rho ^\alpha \varGamma (\alpha n+1)} \left( \lambda a_{n-1}- \mu b_{n-1} \right) . \end{aligned}$$

For $\lambda =\mu =1$ we denote $a_n(\alpha ,\rho )=a_n(\alpha ,\rho ,1,1)$. If in addition $\rho =1$ then $a_n(\alpha )=a_n(\alpha ,1).$

Obviously,

$$\begin{aligned} a_n(1,1,1,1)= \frac{E_n}{2\cdot n!}. \end{aligned}$$

Also,

$$\begin{aligned} a_n(\alpha ,1,1,1)=\frac{E_n^{\alpha }}{2\cdot n!} , \end{aligned}$$

where

$$\begin{aligned} E_n^{\alpha }=\varGamma (\alpha n+1)a_n(\alpha ) \end{aligned}$$

are the Euler fractional numbers introduced in [12] and studied in [3].

We therefore have generalized the Euler numbers $E_n$ and the Euler fractional numbers $E_n^{\alpha }$ to the generalized proportional Euler’s fractional numbers

$$\begin{aligned} E_n^{\alpha ,\rho ,\lambda ,\mu }= \varGamma (\alpha n +1) \cdot a_n(\alpha ,\rho ,\lambda ,\mu ). \end{aligned}$$

## Conclusions

We have introduced a new generalization of the fractional logistic differential equation. To find an explicit solution as a fractional power series, one is lead to the corresponding general fractional Euler’s numbers.

Some figures are plotted to illustrate the results in order to compare the solutions of the classical logistic equation, of the Caputo fractional logistic differential equations and the new generalized proportional fractional logistic differential equation.
